# Oxygen vacancies on Pd/TiO_2_ are detected at low pressures by ESR spectroscopy at ambient temperatures

**DOI:** 10.55730/1300-0527.3416

**Published:** 2022-03-12

**Authors:** Deniz ÜNER, Melis YARAR

**Affiliations:** Chemical Engineering Department, Middle East Technical University, Ankara, Turkey

**Keywords:** Room temperature electron spin resonance (ESR), room temperature electron paramagnetic resonance (EPR), low pressure ESR, saturation-recovery CW-ESR

## Abstract

A low field benchtop electron spin resonance (ESR) (also referred to as electron paramagnetic resonance (EPR)) spectrometer is used to reveal paramagnetic centres such as oxygen vacancies and Ti^+3^ centres over 0.5%Pd/TiO_2_. The measurement was performed at room temperature after the sample was reduced *in situ* under mild hydrogen pressures and evacuated to P < 10^−6^ Torr. The measurement was possible due to a T_1_ compensation effect under vacuum: Correlation times at low pressures enabled sufficient line narrowing and detection of the ESR signal, justifying a method using benchtop spectrometers coupled to vacuum manifolds. The method justification was demonstrated using similar measurements performed on a reference compound, Mn(II) in plasticine: a measurement performed by saturation recovery technique revealed that T_1_ of the signal due to Mn(II) was smaller in vacuum than its atmosphere exposed counterpart. By applying vacuum, the ESR spectra of 0.5%Pd/TiO_2_ were collected at ambient temperatures, with features equivalent to the published data obtained at cryogenic temperatures.

## 1. Introduction

Titanium dioxide can sustain a wide range of stable oxidation states under ambient conditions [1]. The diversity of its oxidation states endows TiO_2_ with rich chemical and catalytic properties. Due to the wide range of stable oxidation states, the material can sustain its crystal structure in the presence of oxygen vacancies: oxygen vacancies can affect the activity of titania by altering the electronic states in the material and creating a charge imbalance. Oxygen vacancies can be created on the TiO_2_ surface by UV irradiation and through thermal treatments such as annealing in vacuum or high temperature and pressure treatment with H_2_ [2–5].

Electron spin resonance (ESR) or also referred to as electron paramagnetic resonance (EPR), (from this point on will be referred to as ESR) is a highly preferred technique to monitor paramagnetic centres or defects such as oxygen vacancy and Ti^+3^ species on titania structure [6–10]. Liu et al. [11], conducted an ESR experiment at 80K to monitor defects introduced to TiO_2_ after H_2_ treatment at different thermal conditions to deduce a relation between hydrogenation temperature and number of defects formed: in particular the amount of Ti^+3^ species was found to be increased with increasing temperature Similarly, Strunk et al. [12] incorporated ESR studies in their work to obtain a concentration of oxygen vacancies after treatment under 10%H_2_/He flow (60cm^3^/min) at 923K for 1 h using ESR signal detected at 12K to conclude that 0.04% Ti was reduced to Ti^+3^ species and ESR signal intensity was linearly correlated to number of oxygens removed. In their work on the effect of heat treatment on the defects formed upon UV-irradiation on titania, Nakaoka and Nosaka [13] performed an ESR experiment under static equilibrium condition supplied by vacuum evacuated and sealed setup at 70K under irradiation to monitor Ti^+3^ species formed. Moreover, Howe and Gratzel [14] also studied UV irradiated colloidal solution of titania, however, they were not able to detect oxygen vacancy or Ti^+3^ species at room temperature but they could see the signal with measurements performed at 77K and 4K. Similarly, Khan et al. [15] were not able to detect any oxygen vacancy or Ti^+3^ ESR signal at room temperature for gray coloured titania sample treated at a cathode chamber of microbial fuel cell, although the species were available to ESR detection at 20K. Recently, Xu et al. [16] showed the oxygen vacancies created in Pd/TiO_2_ structure by Pd catalysed hydrogenation at room temperature and low-pressure hydrogen flow and detected by ESR at 100 K.

As can be seen from the literature review presented, cryogenic temperature studies are widely preferred in literature for ESR studies to provide a high-intensity signal via satisfying the population excess in lower energy state in order to keep transfer from a lower state to the higher state more probable, as indicated by Maxwell-Boltzmann Law [17]. However, cryogenic temperatures are not that readily available for all research purposes and are mostly not compatible with benchtop spectrometers which provide researchers a faster and more practical experiment as well as advanced control over the environment and atmosphere for *in situ* measurements, than its regular-sized counterparts. Hence, here we propose measurements under vacuum as an alternative technique that maintains an external ambient condition while the sample is exposed to conditions equivalent of cryogenic temperature. Similarities of low-temperature and low-pressure procedures are demonstrated and discussed in this work with the reduction of Pd supported titanium dioxide samples.

In this regard, the effect of decreasing pressure to vacuum condition was studied using the synthesis of defective TiO_2_ at ambient conditions with low hydrogen pressure in the presence of Pd. The process was monitored *in situ* by a benchtop electron spin resonance spectrometer at room temperature.

## 2. Experimental

### 2.1. Oxide preparation

0.5%Pd-TiO_2_ was synthesized from Pd precursor (Palladium(II)nitrate(Pd(NO_3_)_2_, 99.9%, Alfa Aesar) and commercial TiO_2_ (P25-Degussa) by incipient wetness technique. An aqueous solution of the Pd precursor was prepared by paying attention to have a sufficient amount of precursor for intended loading and maintaining water amounts to bring about an incipient wetness. All of this solution was impregnated on the preweighed TiO_2_ and the resulting paste was stirred to smoothness. The sample was dried in air overnight and in an oven at 120 °C for 1–2 h, ground, and stored in a dry container.

### 2.2. Electron spin resonance spectroscopy (ESR) analysis

**Figure 1:**
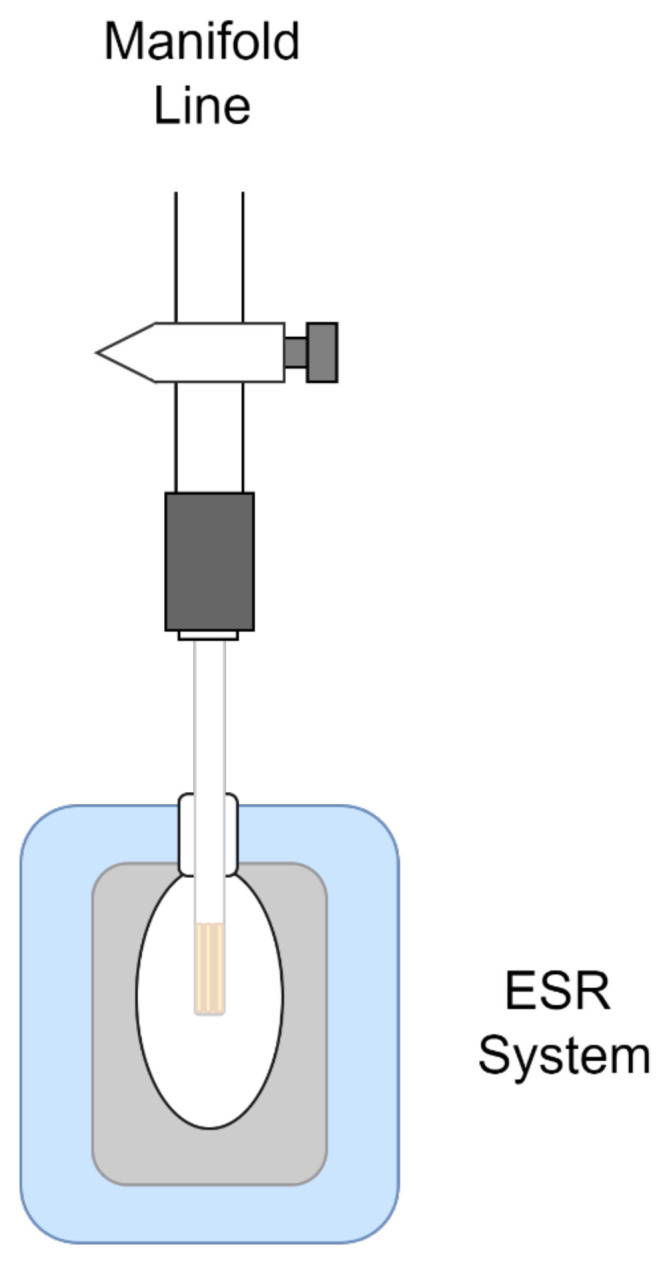
Schematic drawing of ESR system coupled to manifold.

A Benchtop Bruker MicroESR equipment was used to detect oxygen vacancy and Ti^+3^ species formed on 0.5% Pd/TiO_2_. The powder was taken into the ESR tube which was connected to a manifold described in detail elsewhere [18] and schematic drawing given in Figure 1. The manifold components could sustain a background pressure of around 10^−6^ Torr. First, the sample was evacuated fully prior to hydrogen exposure at 120 Torr, supplied to the system at room temperature. The sample was evacuated after a hydrogen exposure at this temperature for 30 min. This procedure was repeated 4 times. Finally, the overnight evacuation was performed. ESR spectra were recorded by maintaining a vacuum *in situ* while 1000 scans were accumulated for a reasonable signal to noise ratio.

Saturation-recovery CW-ESR experiments were conducted using Mn(II) impurity trapped in plasticine as a reference compound. The sample was evacuated and ESR spectra were recorded at all different microwave powers available in Bruker MicroESR. Then, the same procedure was repeated for air-exposed sample at atmospheric pressure. These measurements were used to compare the signal behaviour with the signal obtained from 0.5%Pd/TiO_2_ sample.

## 3. Results and discussion

**Figure 2:**
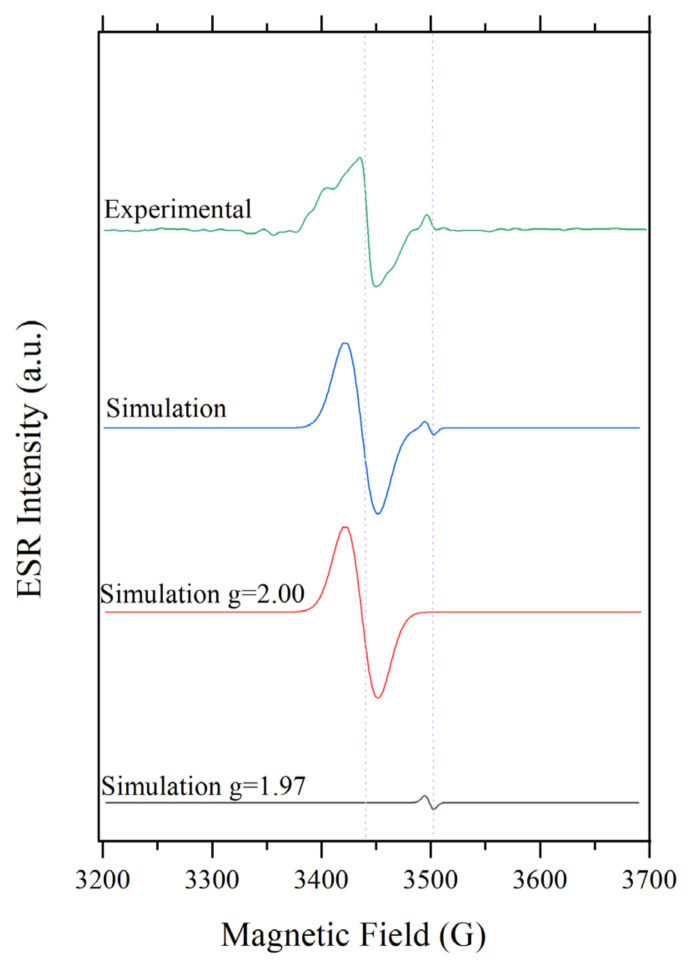
ESR spectrum of 0.5%Pd/TiO_2_ and simulated signals.

Point defect formation on titania surface upon nonpressurized hydrogen treatment was examined by ESR spectroscopy at room temperature. As shown in Figure 2, 0.5%Pd/TiO_2_ sample which was evacuated following the mild-pressure (~0.2 bar), room temperature hydrogen treatment, exhibits paramagnetic behaviour. The experimental signal was simulated with MATLAB Easy-spin ESR simulation tool and simulated peaks are also given in Figure 2. The simulation revealed two different signals: one is at 3500 G with a g-value of 1.97 which is attributed to Ti^+3^ species and a sharp, high-intensity signal at 3445 G indicated the formation of oxygen vacancies with a g-value of 2.00 which is the value for a free electron [4,19–21]. On the contrary, for the untreated 0.5%Pd/TiO_2_ sample and 0.5%Pd/TiO_2_ in the hydrogen environment, no ESR signal was detected. Similarly, the pure TiO_2_ sample was found to be ESR silent in all cases for the experimental conditions available in the benchtop spectrometer. These provided direct proof that, although the pure TiO_2_ cannot, in the presence of Pd it can be reduced under mild hydrogen pressure and temperature. In order to detect a meaningful signal, the system has to be fully evacuated to the base pressure of 10^−6^ Torr. The signal could be detected neither under hydrogen pressure nor exposed to air after reduction.

**Figure 3:**
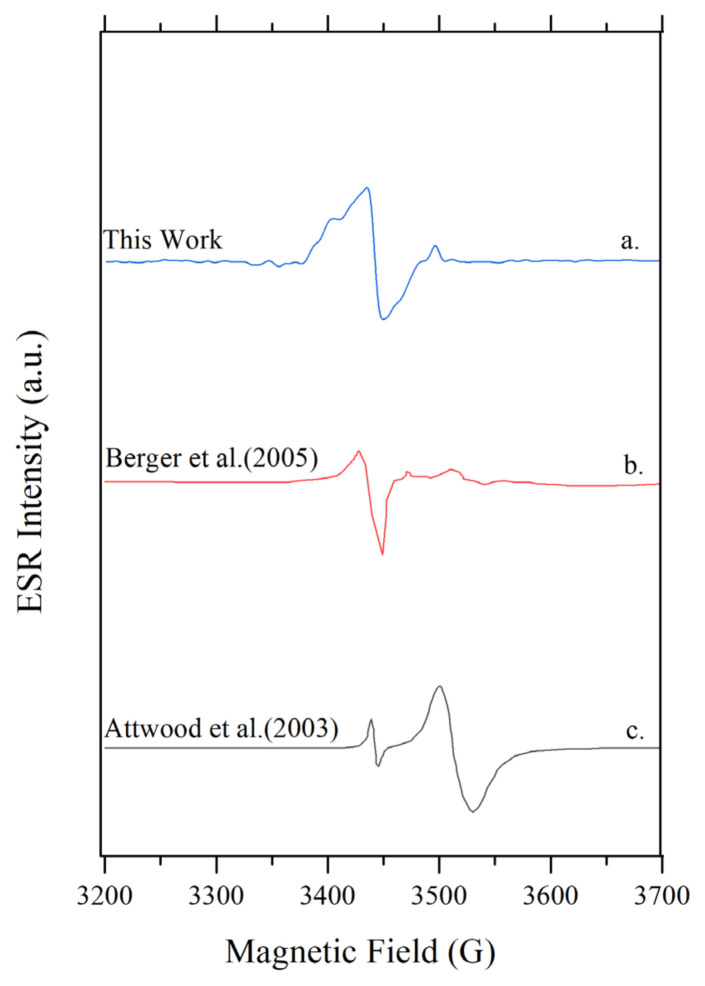
Comparison of the spectrum obtained in this work to literature results **a)** This work **b)** Reproduced from Berger et al. (2005) and **c)** Reproduced from Attwood et al. (2003).

The spectrum presented in this work is similar to the reported spectra in literature obtained from UV treated titania or high temperature/pressure hydrogen treated titania which were measured at cryogenic temperatures. Figure 3 gives the ESR spectrum obtained by Berger et al. [21] after UV irradiation of anatase at 90K. They attributed the first peak with a g-value around 2.00 to O^−^ ions and the second one at g = 1.95 to trapped electrons at Ti^+3^ sites. The spectrum reported by Attwood et al. [20] shows two signals with g = 2.003 and g = 1.962 which were produced by hydrogen exposed reduction at 873K. This spectrum was reported to be obtained at 100K. Both of the two spectra, from Attwood et al. and Berger et al.’s work, explained above exhibit the same signals for oxygen vacancies and Ti^+3^ species reported in this work. The important point to note here is that the data collected from the literature were recorded at cryogenic temperature T < 100 K, while in this work measurements were performed at ambient temperatures albeit under high vacuum. The low signal intensities were compensated by accumulating 1000 signals for better S/N ratios. Hence, by accumulating ESR signals with higher scan counts, while maintaining a dynamic vacuum at ~10^−6^ Torr, a spectrum similar to the ones reported in the literature, could be recorded at room temperature.

The advantage of employing low-temperature procedure is through creating a higher difference between the population densities of the two energy levels described by Maxwell-Boltzmann distribution [17]. In addition to signal intensity enhancement introduced through population difference induced by cryogenic temperatures, spin-lattice relaxation rate is the second factor that affects the quality of the ESR signal [22]. Every spin in the magnetic field experiences local fluctuating magnetic fields which is the source of relaxation. Thermal motion of the surrounding molecules causes these fluctuations and determine the rate of energy dissipation associated with relaxation. The time scale of fluctuations is described by the correlation time, *τ_C_*. As illustrated by Levitt [23], small molecules in nonviscous liquids or gases possess short correlation times and as the temperature decreases correlation time of fluctuations increases due to slower thermal motion of molecules, and hence spin-lattice relaxation time constant T_1_ decreases. As a result, species excited to upper energy level more readily return to lower energy state, increasing the population density dictated by the Maxwell-Boltzmann Law. This behaviour is explained by T_1_ being inversely proportional to the mean transition probability per unit time from one state to the other, i.e. as T_1_ decreases transition probability increases [23]. Consequently, in order to obtain a high-intensity signal satisfying Maxwell-Boltzmann Law, cryogenic temperature ESR experiments are preferred since this way *τ_C_* can be increased by slowing down the thermal motion of molecules and hence causing T_1_ to decrease. In this work, the condition supplied by temperature decrease was created by control of the pressure inside the ESR tube.

Similar to high temperature, at high pressures, molecular fluctuations become more rapid due to shortened path of oscillations. As a result, correlation time of fluctuations, *τ_C_* decrease and for small molecules in gases spin-lattice relaxation time constant, T_1_ increase. This leads to a slower relaxation and a weaker ESR signal. Hence, the contrary strategy, decreasing pressure, should function in the opposite way. As pressure decreases *τ_C_* increases, T_1_ decreases, and the quality of ESR signal is enhanced. The signal presented in this work which was obtained in vacuum condition at room temperature provides direct proof to the phenomenon explained above.

In order to check the validity of this hypothesis, T_1_ values at high pressure and vacuum condition had to be estimated and compared. Saturation-recovery ESR experiment is a way to understand relative spin-lattice relaxation rates using a CW-ESR Spectrometer. The steady-state solutions to the Bloch equations relate the intensity to the microwave power as follows [22]: T_1_ values significantly affect the power dependency of ESR signal intensity, with respect to the equation below, at which I represents intensity, P applied microwave power and b a parameter depending on the line shape:


(1) $$I\propto \frac{\surd P}{{\left(1+\frac{P}{P_{1/2}}\right)}^{b/2}}$$

Where *P*_1/2_ is the power at which the saturation factor given by equation (2) is 1/2


(2) $$P_{1/2}=\left(\frac{1}{\left(1+{\left(\frac{g\;\mu_{B}}{h}\right)}^{2}B_{1}^{2}T_{1}T_{2}\right)}\right)=\frac{1}{2}$$

**Figure 4:**
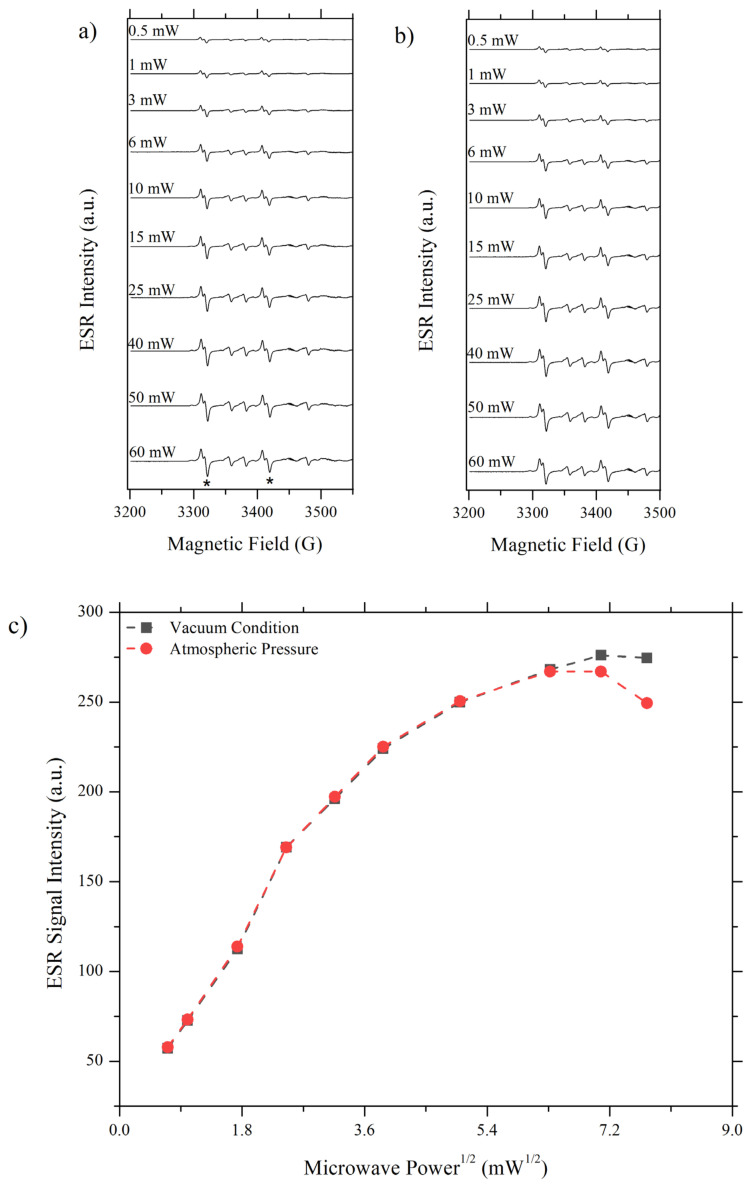
Saturation-recovery CW-ESR Experiment **a)** Mn(II)/Plasticine in vacuum condition **b)** Mn(II)/plasticine in atmospheric pressure **c)** Signal intensity vs. microwave power^1/2^ analysis for atmosphere exposed and evacuated Mn(II)/plasticine.

Microwave power is absorbed by sample function in a way that population difference between higher and lower states decreased as more microwave is absorbed. On the contrary, the spin-lattice relaxation process works in a conflicting manner to increase population difference. If microwave power reaches a value that is too high for spin-lattice relaxation to oppose, saturation phenomena occur and the signal becomes broader and low intensity [24]. In order to obtain a high-intensity signal, microwave power has to be optimised such that microwave-driven transition to upper state and spin-lattice relaxation induced returns to lower state are balanced. This power and T_1_-dependent nature of ESR signal let one measure spin-lattice relaxation rate by employing saturation-recovery ESR experiment. In such an experiment, a power value that signal can be detected is selected and changed until signal intensity decreases [25]. Since in this work it is desired to compare T_1_’s in air and in a vacuum and since the signal for Pd/TiO_2_ cannot be detected under oxygen atmosphere, a standard sample, Mn(II) impurity in plasticine, was used. The results of the saturation-recovery experiment on this sample are given in Figure 4.

In Figures 4.a and 4.b, ESR signals obtained at different microwave power values for atmospheric pressure and in vacuum conditions can be seen. Two detected hyperfine sextet lines were indicated with *, it was not possible to detect the rest of the hyperfine sextets due to limitations on the frequency range of the spectrometer used. In Figure 4.c, signal intensities of two distinct atmospheres were compared with respect to the square root of microwave power using the signal at 3311 G as reference. The comparison revealed that 7.07 mW^1/2^ corresponding to 50 mW is the optimum value for microwave power at which the microwave-induced transitions and spin-lattice relaxation is balanced. If the power is decreased or increased from this value signal intensity decreases for both conditions. However, at high microwave power, a change in signal intensity is more significant for the sample measured at atmospheric pressure than for the evacuated sample. Since air-exposed sample is more affected by the increase in power, it can be concluded that the spin-lattice relaxation rate is too slow to oppose microwave-induced transition, i.e. T_1_ at atmospheric pressure is larger than in vacuum, indicating the pressure dependency of T_1_.

**Figure 5:**
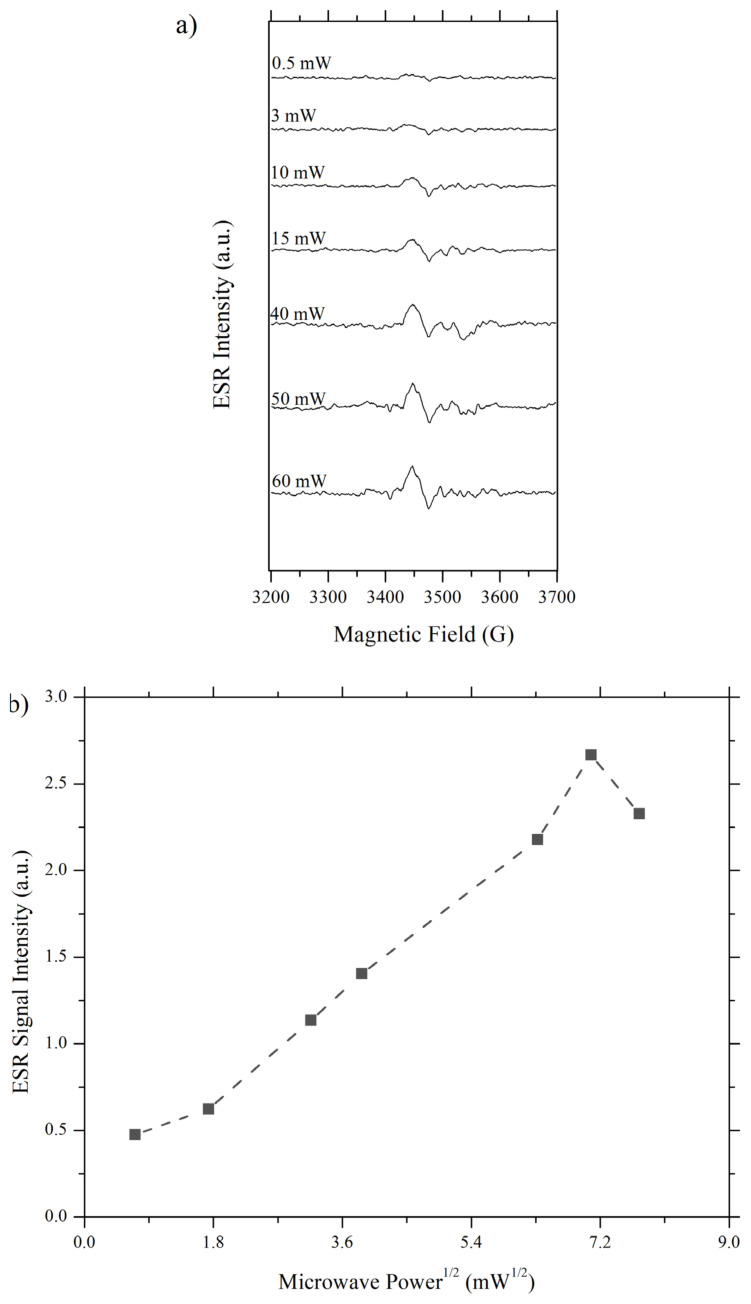
Saturation-recovery CW-ESR experimental results for 0.5%Pd/TiO_2_ **a)** Spectrum **b)** Signal intensity vs microwave power^1/2^ analysis.

For 0.5%Pd/TiO_2_ sample, saturation-recovery experiment was only possible under vacuum condition. As seen in Figure 5, the optimum signal was recorded at P^1/2^ = 7.07 mW^1/2^ corresponding to 50 mW microwave power, revealing that T_1_ dependent nature of power for this sample follows the same principles as reference sample. However, the signal intensity in Pd/TiO_2_ is much lower than plasticine, dependency on power is sharper and additionally at low power values signal almost disappears.

In literature ultra-high vacuum (UHV) ESR spectrometry is a frequently used method. However, to the best of authors’ knowledge, the reported reason for employing high vacuum was to prevent the destruction of paramagnetic centres by chemisorption of adsorbing species [e.g., 26,27]. If this explanation was valid for the saturation-recovery experimental results presented in this work, signal intensity under air exposed sample had to be smaller than the evacuated sample for any microwave power value. However, for power values smaller than 50 mW, the signal intensities in both cases are identical. The change in power-dependent behaviour of intensity is only observed at microwave powers higher than 50 mW. The ultimate reason for that; the signal is not affected by adsorbing species, but only by competition between spin-lattice relaxation and microwave-induced transition. Hence, here we suggest: low-pressure strategy provides the conditions supplied by the low-temperature method as both adjust the spin-lattice relaxation time constant through regulating the correlation time of magnetic field fluctuations induced by the thermal motions of the molecules.

## 4. Conclusion

It was concluded that defects on the titania surface can be synthesized under mild hydrogen pressure at ambient temperature in the presence of Pd and can be detected by a benchtop ESR spectrometer at room temperature with *in situ* experiments in vacuum conditions. This is due to the low-pressure method supplying the same conditions as the low-temperature one by regulating the correlation time of fluctuations of magnetic field experienced by the spins and hence adjusting the relaxation rate.
